# Comparison of multiple doses of corticosteroids in Kawasaki disease: a Bayesian network analysis

**DOI:** 10.3389/fphar.2025.1661380

**Published:** 2025-10-27

**Authors:** Xuan Li, Xuan Tang, Daoping Yang, Miao Hou, Qiuqin Xu, Yunjia Tang, Bo Wang, Hongbiao Huang, Ye Chen, Zhiheng Liu, Guanghui Qian, Haitao Lv

**Affiliations:** ^1^ Department of Cardiology, Children’s Hospital of Soochow University, Suzhou, China; ^2^ Department of Pediatrics, Institute of Pediatric Research, Jiangyin People’s Hospital, Wuxi, China; ^3^ Institute of Pediatric Research, Children’s Hospital of Soochow University, Suzhou, China

**Keywords:** Kawasaki disease, corticosteroid, Bayesian network analysis, coronary artery lesions, immunoglobulin resistance

## Abstract

**Background:**

Kawasaki disease (KD) is a leading cause of acquired heart disease in children, with coronary artery lesion (CAL) as a major complication. Although intravenous immunoglobulin (IVIG) remains the cornerstone of therapy, corticosteroids continue to play an important role in the management of IVIG-resistant, high-risk, or severe Kawasaki disease. Nevertheless, the optimal dosing strategies and differential therapeutic effects of corticosteroids in children with distinct clinical subtypes of KD remain poorly understood, particularly in those at highest risk.

**Methods:**

We conducted a Bayesian network meta-analysis of five regimens: intravenous immunoglobulin alone (IVIG-alone), medium-dose methylprednisolone alone (MDMP-alone), high-dose methylprednisolone alone (HDMP-alone), IVIG-plus-low-dose methylprednisolone (IVIG-plus-LDP), and IVIG-plus-HDMP. Data from randomized controlled trials (RCTs) through December 2024 were included.

**Results:**

IVIG-plus-HDMP ranked highest for preventing treatment resistance and reducing fever in initial and refractory KD [Surface Under the Cumulative Ranking Curve (SUCRA) 0.79]. IVIG-plus-LDP had the highest probability of reducing coronary artery dilation (CAD) incidence (SUCRA 0.89). Corticosteroid-related side effects (e.g., bradycardia, hypertension) were mild, transient, and reversible across all regimens, with no severe adverse events reported.

**Conclusion:**

IVIG-plus-HDMP is the most effective therapy for acute symptom control in KD, particularly in high-risk or IVIG-resistant cases, while IVIG-plus-LDP appears superior for long-term prevention of coronary complications in the general KD population. Treatment selection should be individualized based on patient risk profile and treatment priorities, balancing rapid symptom management against long-term coronary outcomes.

**Systematic Review Registration:**

https://www.crd.york.ac.uk/prospero/, identifier CRD42022339937.

## 1 Introduction

Kawasaki Disease (KD) is a systemic vasculitis predominantly affecting children under 5 years of age (Rowley and Shulman, 2018), represents the leading cause of acquired cardiovascular disease in children (Kainth and Shah, 2019). Its incidence has shown a concerning upward trend in recent years (Fukazawa et al., 2020), with coronary artery lesions (CALs) constituting its most severe complication (Zhang et al., 2020). Indeed, KD is now recognized as the foremost cause of acquired heart disease among children in developed countries (McCrindle et al., 2017).

The current standard therapy for KD combines high-dose intravenous immunoglobulin (IVIG) with aspirin. Despite this regimen, CALs persist in approximately 4% of patients (Zheng et al., 2021), and IVIG resistance occurs in 10%–15% (Chen et al., 2022). Corticosteroids have emerged as a potential adjunctive therapy, both in the initial phase and for IVIG-resistant KD. Studies suggest they may reduce CALs incidence (Chen et al., 2016) and effectively alleviate fever in IVIG-resistant patients without increasing CALs risk (Furukawa et al., 2008). However, a dose-dependent increase in adverse effects has also been reported (Kauh et al., 2012). While intravenous immunoglobulin (IVIG) is the mainstay of therapy, corticosteroids remain essential in managing IVIG-resistant, high-risk, or severe Kawasaki disease. However, their optimal dosing and efficacy across different clinical subtypes in children are not yet well defined.

While traditional pairwise meta-analyses (Chen et al., 2016; Zhu et al., 2012; Yang et al., 2018; Green et al., 2022) have compared corticosteroids to IVIG regarding antipyretic efficacy and impact on CALs, while none have evaluated the effectiveness of different doses of steroids. Pairwise meta-analyses are inherently limited to direct comparisons between two interventions.

Network meta-analysis (NMA) overcomes this limitation by enabling simultaneous comparisons of multiple interventions (Tromp et al., 2022; Giacoppo et al., 2024). Both Bayesian and frequentist frameworks are applicable; however, we employed a Bayesian approach for its distinct advantages in this context: its capacity to incorporate prior knowledge, provide a more comprehensive quantification of uncertainty (particularly valuable with sparse data), and offer direct probabilistic interpretations of treatment rankings—features especially pertinent to complex clinical decision-making.

Therefore, this study aimed to utilize Bayesian network meta-analysis to compare the effectiveness of three distinct corticosteroid doses in children with KD, specifically evaluating their impact on: antipyretic effect, Reduction of coronary artery dilation (CAD), and Incidence of adverse effects.

## 2 Methods

### 2.1 Study design

This NMA was conducted in accordance with the PRISMA Extension Statement for Network Meta-Analyses (PRISMA-NMA) and the Cochrane Handbook for Systematic Reviews of Interventions (Higgins JP, 2011; Hutton et al., 2015).

### 2.2 Literature search strategy

A systematic search was performed in the following electronic databases from inception to 31 December 2024: Cochrane Central Register of Controlled Trials, EMBASE, and PubMed. Search terms included controlled vocabulary (e.g., MeSH terms) and keywords related to: “Kawasaki Disease,” “Mucocutaneous Lymph Node Syndrome,” and “Steroids,” “Methylprednisolone,” “Prednisolone,” “Corticosteroids,” “Glucocorticoids,” and “Randomized Controlled Trial.” Additional records were identified through Hand-searching reference lists of included studies and relevant systematic reviews. The systematic search strategy and search terms were documented in the Supplementary Appendix.

### 2.3 Eligibility criteria and data abstraction

Studies were included based on the following criteria: 1) all patients were under 18 years old; 2) diagnostic criteria for KD were based on Japanese diagnostic criteria or the American Heart Association common standards. According to the criteria set by the Japanese Ministry of Health (Group, 2014), CAD were defined as having an internal lumen diameter greater than 3 mm in children younger than 5 years or greater than 4 mm in children 5 years or older. In the z-score system (McCrindle et al., 2017), CAD were defined as having a Z-score greater than 2.0 after one or 2 month of onset; 3) All patients were treated with IVIG; 4) IVIG-resistance was defined as persistent or recrudescent fever of body temperature ≥38.0 °C at least 36 h after completion of the first IVIG infusion; 5) the effectiveness of treatment was defined as defervescence after treatment; 6) patients received corticosteroid either in initial or refractory KD treatment; 7) studies were written in English. Studies that did not meet these criteria were excluded from the analysis. Data extracted from each study included demographic characteristics (e.g., age), type of treatment, treatment time, outcomes (e.g., CAD, side effects), and study location.

To ensure accuracy, relevant information was independently reviewed and extracted from publications by two authors (XL and TX), and discrepancies were resolved through discussion or with the involvement of a third author (LHT).

### 2.4 Outcome measures

The summary data and demographic characteristics of each study were analyzed, and the effectiveness of treatment was identified as the primary outcomes. The effectiveness of treatment was defined as defervescence after treatment. The secondary outcomes included the development of CAD, and side effects. In addition, the authors of relevant studies were contacted to obtain missing results. Studies that did not respond to the request within a month were excluded from the analysis.

### 2.5 Risk of bias (ROB) and quality assessment

The Cochrane Risk of Bias Tool (Higgins JPT et al., 2019) was applied to assess the risk of bias in the included randomized controlled trials (RCTs), as assessed by MH and DPY.

### 2.6 Statistical analyses

A connected network integrating direct and indirect evidence was formed using the Bayesian statistical model to compare different doses of corticosteroids (Tonin et al., 2017). Data were extracted from each study and direct and indirect comparisons were compared using the random effects model. Model parameters obtained after four Markov chains with over-dispersed values and Gibbs sampling based on 50,000 iterations., with a “burn-in” period of 20,000 simulations set. Model convergence was assessed using the Brooks-Gelman-Rubin diagnosis plots method, trace plot, and density plot (Brooks and Gelman, 1998). The main analysis of therapy effectiveness employed the default priors of the gemtc package. While the analysis of CAD explicitly used a weakly informative uniform prior for the heterogeneity parameter: sd.d ∼ dunif(0, 2).

Corticosteroid therapy effectiveness, CAD incidence were measured using odds ratio (OR) with 95% credible interval (CI). A Bayesian approach was utilized to estimate OR along with their associated 95% CI, providing a probabilistic interpretation of the effectiveness of each intervention. This approach calculates the posterior distributions of ORs, allowing for a direct comparison between treatments while accounting for the uncertainty in the estimates. The surface under the cumulative ranking curve (SUCRA) values were then derived from these posterior distributions, summarizing the relative ranking of each treatment option based on its probability of being the most effective, with a SUCRA value of 1 represents the most favorable outcome and 0 represents the least favorable outcome.

Sensitivity analyses were performed by re-running both models using alternative weakly informative priors for the heterogeneity parameter (τ), including a Half-Normal(0, 1) prior—implemented as τ ∼ Normal(0, 1)I(0,)—and a Uniform(0, 5) prior to evaluate the impact of a less restrictive constraint on heterogeneity. Additionally, a pre-planned sensitivity analysis was conducted by excluding the study Ogata et al. (2012) that exclusively enrolled patients predicted to be IVIG-resistance to assess its unique influence on the overall results.

Subgroup analysis was conducted based on the onset time of corticosteroid treatment. The initial treatment was defined as the administration of corticosteroids as the primary treatment. We defined retreatment as the administration of corticosteroids for IVIG-resistant KD.

The analyses were performed in WinBugs 14 or R language (4.2.1 version)) by using both “Gemtc” (version: 1.0-1) and “rjags” (version: 4-13). Comparison-adjusted funnel plots under the random effect model were used to visually examine publication bias. These statistical analyses were performed using Stata 16.0 (StataCorp LLC, College Station, TX 77845). The risk of bias estimation was done using Review Manager 5.4 software (The Nordic Cochrane Centre, Copenhagen, Denmark).

## 3 Results

### 3.1 Literature search and study characteristics

**FIGURE 1:**
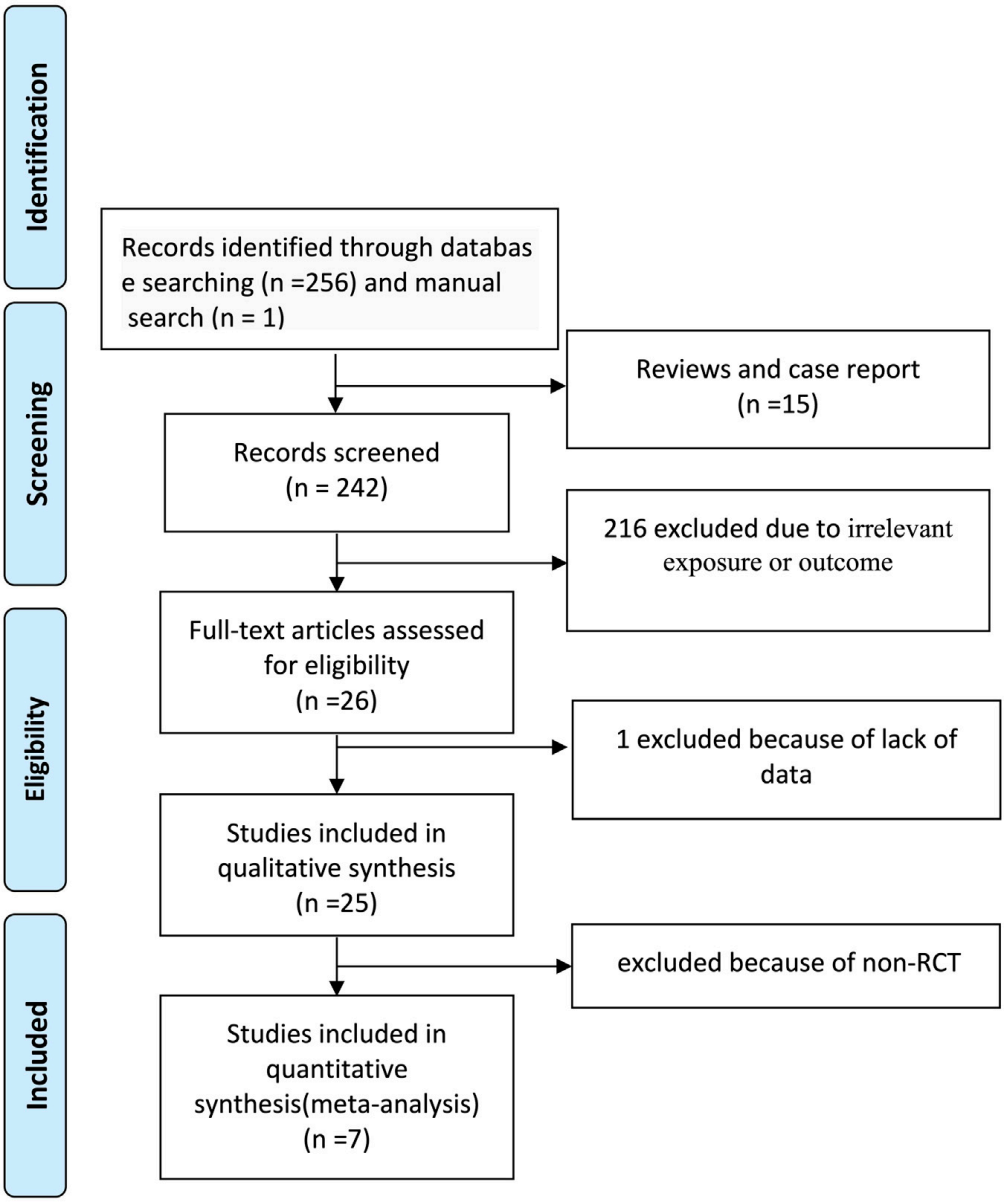
Flow diagram of study inclusion.

The systematic literature search yielded 257 records. After exclusion of 231 records (15 reviews/case reports and 216 irrelevant studies), 26 full-text articles underwent eligibility assessment. Nineteen studies were excluded: one due to insufficient data and 18 as non-randomized trials. Seven RCTs (Miura et al., 2008; Wang et al., 2020; Inoue et al., 2006; Sundel et al., 2003; Kobayashi et al., 2012; Ogata et al., 2012; Newburger et al., 2007) involving 801 patients were included in the final network meta-analysis (Figure 1).

Corticosteroid regimens were categorized into three predefined doses based on reported protocols. Low-dose (LD): Prednisolone 2 mg/kg/day; medium-dose (MD): Methylprednisolone 15 mg/kg/day; high-dose (HD): Methylprednisolone 30 mg/kg/day (pulse therapy). The comparator group received IVIG without corticosteroids. Five treatment nodes formed the evidence network: IVIG-alone group (standard IVIG therapy without corticosteroids), MDMP-alone group (medium-dose methylprednisolone [MDMP] monotherapy), HDMP-alone group (high-dose methylprednisolone [HDMP] monotherapy), IVIG-plus-LDP group (IVIG combined with low-dose prednisolone[LDP]), IVIG-plus-HDMP group (IVIG combined with HDMP).

Geographically, studies originated from Japan (n = 4), the United States (n = 2), and China (n = 1). Five trials evaluated initial KD therapy (IVIG combined with corticosteroids), while two focused on IVIG-resistant KD (monotherapy comparisons). Detailed study characteristics are presented in Table 1.

**TABLE 1:** Characteristics of the studies included in the analysis.

Author	Country	Multicenter	No. of patients in treatment/control group	Male (treatment/control)	Patients age (treatment/control) (year)	Initial treatment (treatment/control)	Retreatment (treatment/control)
Wang et al. (2020)	China	No	40/40	28/28	2.1 ± 1.8/1.8 ± 1.2	IVIG	MDMP-alone group/IVIG-alone group
Miura et al. (2008)	Japan	No	7/8	5/5	2.7 ± 1.6/2.6 ± 2.2	IVIG	HDMP-alone group/IVIG-alone group
Inoue et al. (2006)	Japan	Yes	90/88	51/51	2.4 ± 1.9/2.3 ± 1.7	IVIG-plus-LDP/IVIG-alone group	
Sundel et al. (2003)	USA	No	18/21	12/15	4.5(0.4–13.5)/4.3(0.6–12.3)	IVIG-plus-HDMP/IVIG-alone group	
Kobayashi et al. (2012)	Japan	Yes	121/121	67/68	2.9(1.0–4.2)/2.5(1.1–3.9)	IVIG-plus-LDP/IVIG-alone group	
Ogata et al. (2012)	Japan	No	22/26	11/13	3.4 ± 0.2/3.5 ± 0.6	IVIG-plus-HDMP/IVIG-alone group	
Newburger et al. (2007)	USA	Yes	101/98	62/62	2.9(1.3–5.0)/2.9(1.6–4.4)	IVIG-plus-HDMP/IVIG-alone group	

RCT, randomized controlled trial; LDP, low-dose prednisolone; MDMP, medium-dose methylprednisolone; HDMP, high-dose methylprednisolone.

### 3.2 Risk of bias and publication bias

**FIGURE 2:**
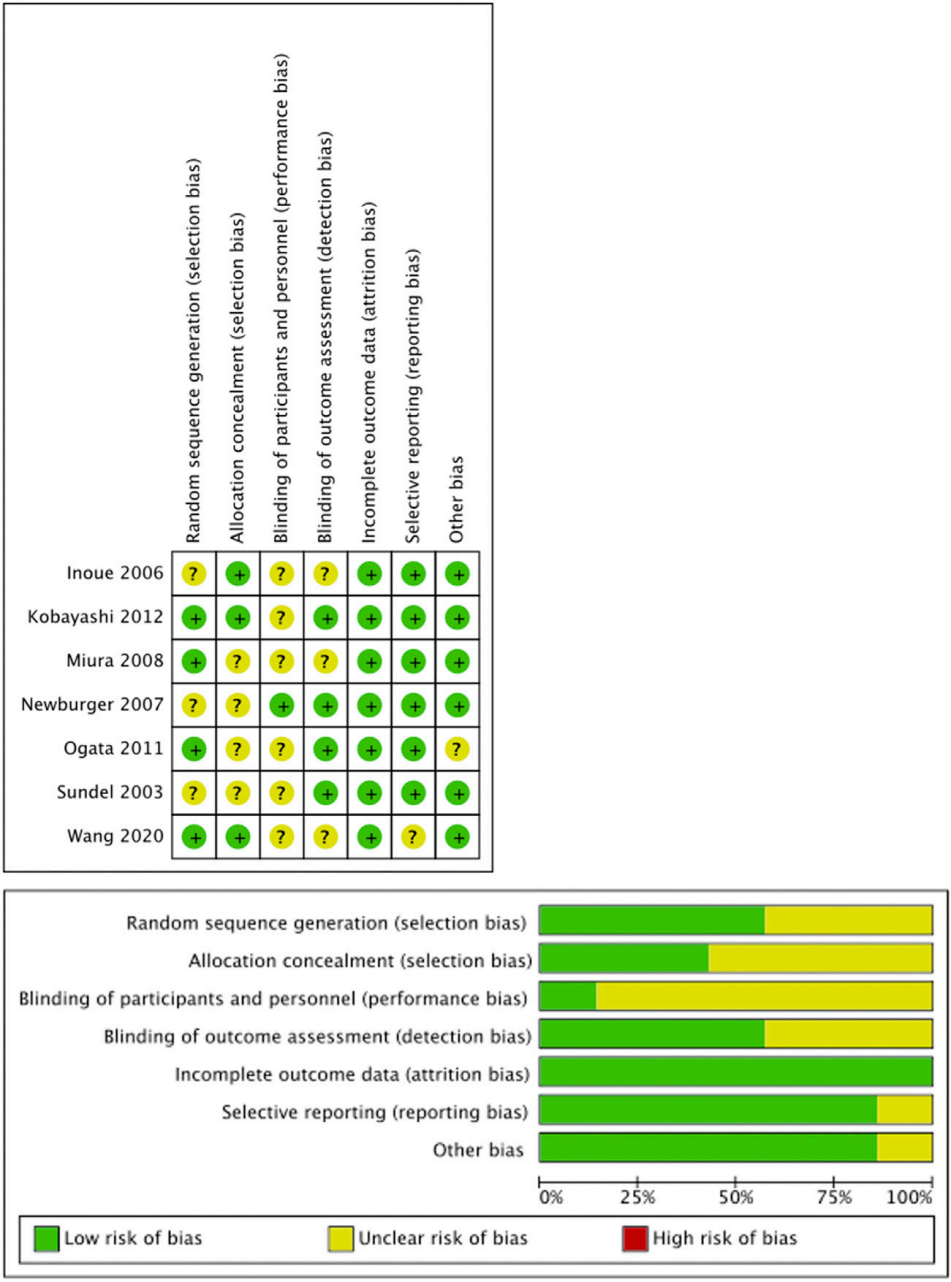
Risk of bias assessment for RCT studies.

**FIGURE 3:**
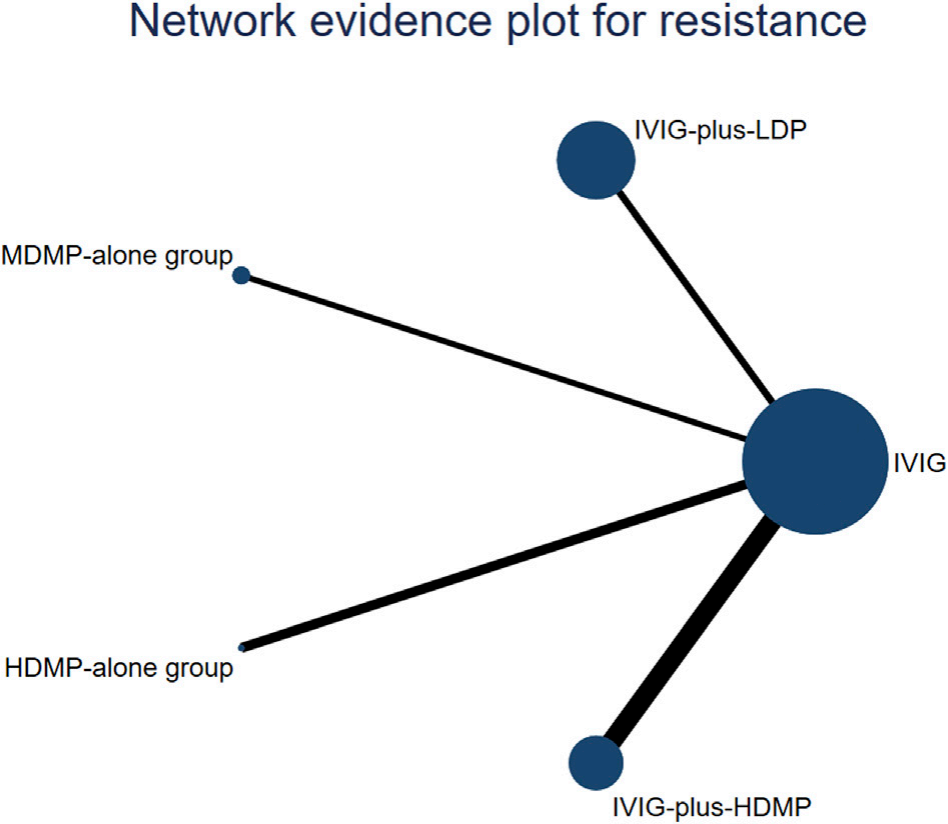
Network constructions. The circle size represents the sample size of the group. The size of each circle represents the number of patients in each treatment node. The connecting lines represent direct comparisons in the studies. The width of the lines connecting different treatment nodes reflects the number of studies available for each comparison.

All included RCTs demonstrated low risk of bias across Cochrane RoB 2.0 domains (Figure 2). Comparison-adjusted funnel plots showed symmetrical distribution, indicating low substantial publication bias (Figure 3).

### 3.3 Network meta-analysis for effectiveness (defervescence)

**FIGURE 4:**
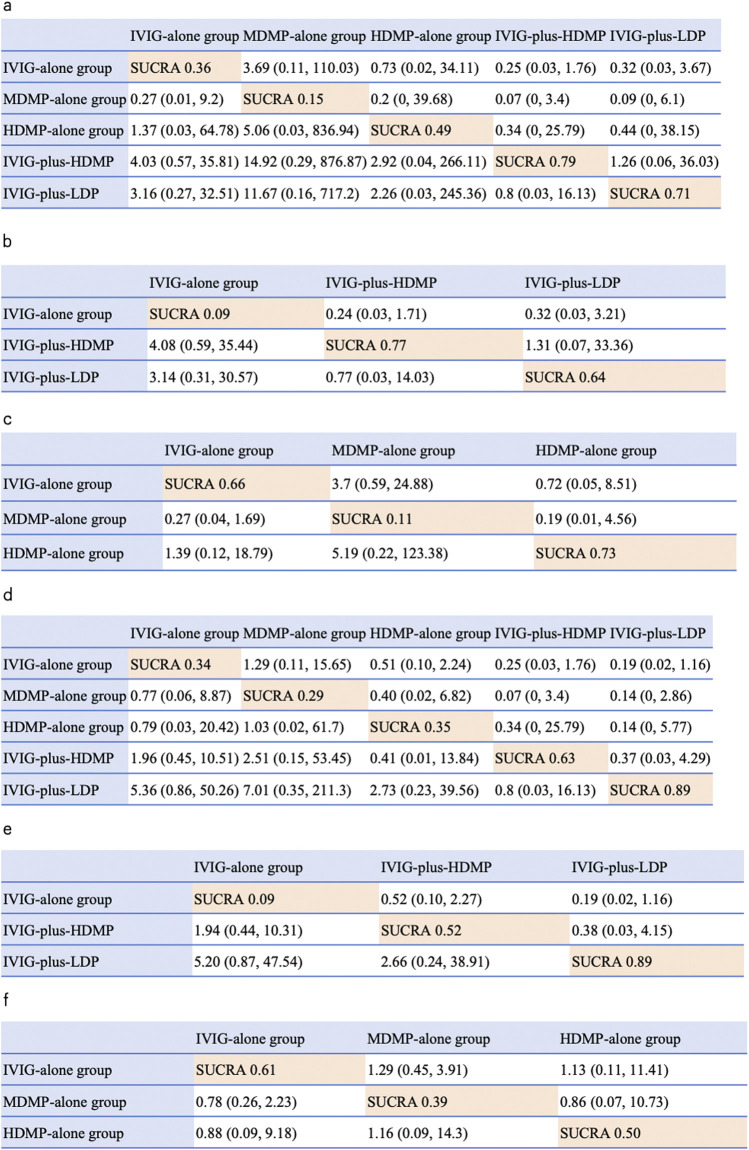
NMA results of effectiveness and coronary artery abnormality, followed by the ranking distribution according to SUCRA values. The highest SUCRA values indicate the highest probability of being the most effective treatment, and the lowest probability of the incidence of coronary artery dilation (CAD). Numbers in orange boxes were SUCRA values, where a value of 1 represents the most favorable outcome and 0 represents the least favorable outcome. The white square shows the ORs (Column vs. Row). **(a)** NMA results of treatment effectiveness, **(b)** NMA results of treatment effectiveness in initial stage treatment. **(c)** NMA results of treatment effectiveness in refractory stage treatment. **(d)** NMA results of the incidence of CAD. **(e)** NMA results of CAD in initial stage treatment. **(f)** NMA results of CAD in refractory stage treatment.

**FIGURE 5:**
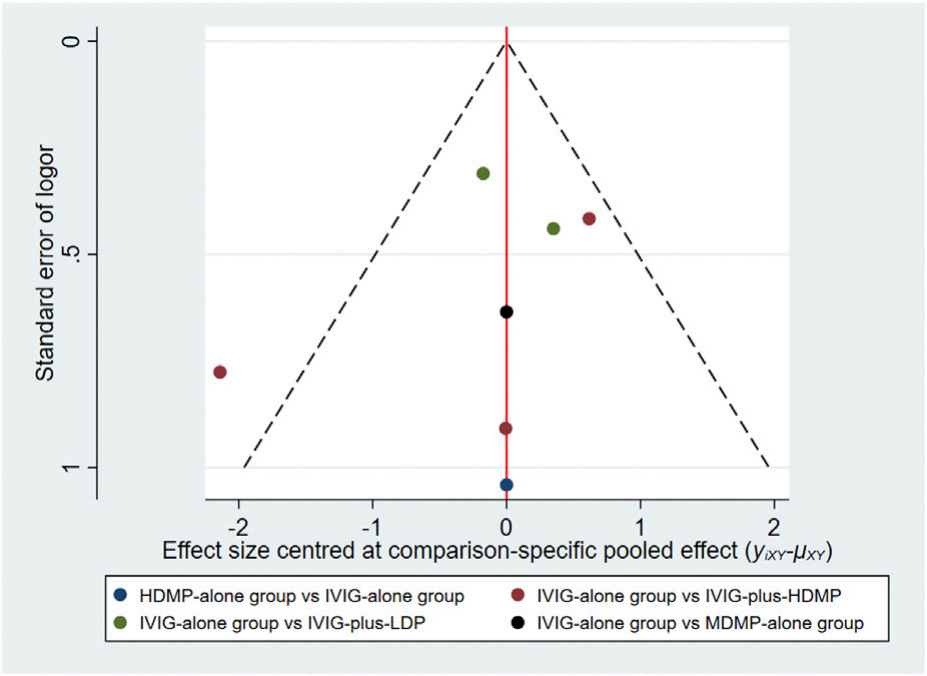
Network funnel plot for comparison of treatment effectiveness. Each circle represents a individual study contributing to the comparison; the outlier red circle denotes Ogata et al. (2012), the upper red circle indicates Newburger et al. (2007), the lower red circle represents Sundel et al. (2003), the upper green circle corresponds to Kobayashi et al. (2012) and the lower green circle to Inoue et al. (2006). The blue circle represents Miura et al. (2008), and the black circle denotes Wang et al. (2020).

The network geometry incorporated all five treatments across seven studies (Figure 4). In Bayesian random-effects models, IVIG-plus-HDMP group showed a non-significant trend toward reduced treatment failure versus IVIG alone group (OR 4.03, 95% CrI 0.57–35.81), as did IVIG-plus-LDP group (OR 3.16, 95% CI 0.27–32.51). SUCRA ranking indicated IVIG-plus-HDMP group had the highest probability of being the most effective intervention (SUCRA 0.79), followed by IVIG-plus-LDP group (0.71), HDMP-alone group (0.49), IVIG-alone group (0.36), and MDMP-alone group (0.15) (Figure 5a). Subgroup analyses revealed: for initial KD, IVIG-plus-HDMP group ranked highest (SUCRA 0.77) (Figure 5b); for IVIG-resistant KD, HDMP-alone group ranked highest (SUCRA 0.73) (Figure 5c).

### 3.4 Network meta-analysis for the incidence of CAD

IVIG-plus-LDP group demonstrated the lowest probability of CAD occurrence (SUCRA 0.89), indicating superior preventive efficacy. Subsequent rankings were IVIG-plus-HDMP group (SUCRA 0.63), HDMP-alone group (SUCRA 0.35), IVIG-alone group (SUCRA 0.34), and MDMP-alone group (SUCRA 0.29) (Figure 5d). Subgroup analyses stratified by treatment timing showed: in initial KD therapy, IVIG-plus-LDP group maintained optimal CAD prevention (SUCRA 0.89) (Figure 5e); in IVIG-resistant KD, IVIG alone group ranked most favorably (SUCRA 0.61) (Figure 5f).

### 3.5 Treatment-related adverse events

Adverse event data were available from 6 of the 7 included studies (Table 2). The most frequently reported events were hypertension (n = 12, six cases of hypertension in HDMP-alone group, five cases of hypertension in IVIG-alone group, one cases of hypertension in IVIG-plus-HDMP group,) and bradycardia (n = 13, Five cases of bradycardia in MDMP-alone group, six cases of bradycardia in HDMP-alone group, two cases of bradycardia in IVIG-plus-HDMP group), with no severe sequelae documented. All events resolved spontaneously with supportive care. Due to heterogeneity in reporting methods and insufficient comparative data across treatment nodes, formal network meta-analysis for adverse events was not feasible.

**TABLE 2:** Side effect reported in included studies.

Study	Side effect
Wang et al. (2020)	Five cases of bradycardia in MDMP-alone group
Miura et al. (2008)	Six cases of bradycardia in HDMP-alone group, two cases in IVIG-alone group, five cases of hyperglycemia in HDMP-alone group, one case of hypothermia in HDMP-alone group, six cases of hypertension in HDMP-alone group, five cases of hypertension in IVIG-alone group
Sundel et al. (2003)	Four cases of side effect (including headache, rogors, vomiting, congestive heart failure) in IVIG-plus-HDMP group, twelve cases of side effect in IVIG-plus-HDMP; one cases of hypertension in IVIG-plus-HDMP group
Kobayashi et al. (2012)	High total cholesterol in two patients (10.4 mmol/L and 12.0 mmol/L) and neutropenia in one patient (300/μL) in IVIG-plus-LDP group, high total cholesterol (11.8 mmol/L) and a non-occlusive thrombus in the left coronary artery on echocardiography in IVIG-alone group
Ogata et al. (2012)	Six cases of hypothermia and two cases of bradycardia in IVIG-plus-HDMP group
Newburger et al. (2007)	Five cases of hypotension in IVIG-plus-HDMP group, one case of hypotension in IVIG-alone group

### 3.6 Sensitivity analysis

The key findings from the main and sensitivity analyses are summarized in Table 3 below for the outcome, with consistent results. The results remained consistent across different weakly informative prior distributions for the heterogeneity parameter, including Half-Normal(0,1) and Uniform(0,5) priors, indicating the robustness of the outcomes to prior specifications. Furthermore, A sensitivity analysis was conducted by excluding the study by Ogata et al. (2012), which uniquely enrolled patients predicted to have refractory KD based on the Egami score. This exclusion led to some fluctuations in the SUCRA rankings. Most notably, for the outcome of resistance, the SUCRA value for IVIG-plus-LDP increased from 0.71 to 0.88, while that for IVIG-plus-HDMP decreased from 0.79 to 0.62.

**TABLE 3:** Results of sensitivity analysis: comparison of heterogeneity and treatment ranking (SUCRA values) under different prior specifications in the Bayesian random-effects model.

Parameter	CAL				Resistance			
	Main analysis	Half-Normal prior	Uniform prior	Sensitivity (excl. Ogata et al. (2012))	Main analysis	Half-Normal prior	Uniform prior	Sensitivity (excl. Ogata et al. (2012))
Heterogeneity (τ)	0.89	0.7	0.88	0.79	0.31	0.44	1.15	0.32
SUCRA: IVIG-alone group	0.34	0.33	0.34	0.40	0.36	0.37	0.35	0.38
SUCRA: MDMP-alone group	0.29	0.28	0.29	0.32	0.15	0.18	0.12	0.07
SUCRA: HDMP-alone group	0.35	0.34	0.35	0.38	0.49	0.49	0.49	0.55
SUCRA: IVIG-plus-HDMP	0.63	0.64	0.63	0.48	0.79	0.77	0.81	0.62
SUCRA: IVIG-plus-LDP	0.89	0.91	0.89	0.92	0.71	0.69	0.73	0.88

## 4 Discussion

Our network meta-analysis comprehensively evaluated the efficacy of various corticosteroid regimens in Kawasaki disease, with a focus on both acute symptom management and the prevention of coronary artery complications. The results demonstrate that IVIG-plus-HDMP was most effective for rapid fever reduction in acute KD, whereas IVIG-plus-LDP showed superior efficacy in preventing coronary artery lesions compared to both IVIG-plus-HDMP and IVIG alone. In patients with refractory KD, HDMP monotherapy was most effective for fever control, while IVIG monotherapy remained important for coronary protection. Critically, corticosteroid monotherapy did not surpass IVIG monotherapy in preventing CAD, reaffirming the foundational role of IVIG in KD management. These findings suggest a context-dependent treatment approach: IVIG-plus-HDMP may be preferred for acute symptom control, while IVIG-plus-LDP offers an optimal benefit-risk profile for long-term coronary protection. Importantly, treatment hierarchy appears modulated by IVIG responsiveness, indicating that high-risk patients may benefit from more intensive steroid strategies, whereas IVIG-plus-LDP emerges as the most effective regimen for the general KD population in reducing both resistance and CAD risk. These insights warrant further validation in prospectively stratified trials.

Corticosteroids, widely used for their anti-inflammatory properties, suppress nuclear factor kappa B activity, making them effective for inflammation-driven conditions like KD (Hudson et al., 2018). Prior studies have shown that corticosteroids rapidly reduce fever in initial KD treatment (Chen et al., 2016) and were effective in IVIG-refractory cases (Sosa et al., 2019). Ogata et al. (Ogata et al., 2009) reported that IVIG plus methylprednisolone inhibits inflammatory cell transcripts, including those from monocytes, macrophages, and T cells, reducing KD-related cytokine production and IVIG-refractory factors (Inoue et al., 2020a; Inoue et al., 2020b). Our analysis supports these findings, demonstrating that IVIG-plus-HDMP reduces treatment failure rates compared to other therapies. In refractory KD, HDMP-alone therapy showed a stronger anti-inflammatory effect than corticosteroid monotherapy, reinforcing the role of corticosteroids in KD management.

Conflicting evidence exists regarding corticosteroids’ impact on CAD. Newburger et al. (Newburger et al., 2007) found no significant difference in CAD incidence between IVIG and corticosteroid groups, whereas Kobayashi et al. (Kobayashi et al., 2009) reported improved coronary outcomes with IVIG plus corticosteroids. A meta-analysis by Green et al. (Green et al., 2022) showed that corticosteroids, as first or second-line therapy, reduced CAD incidence, inflammatory markers, and hospital stay duration without notable adverse events. However, this study did not compare corticosteroid doses. Our SUCRA analysis indicated that IVIG-plus-LDP therapy was superior to IVIG-plus-HDMP therapy and IVIG-alone therapy in reducing CAD, despite no statistical difference due to low CAD incidence. These results suggest that IVIG-plus-LDP may benefit patients with existing CAD or high CAD risk, potentially avoiding the need for high-dose corticosteroids. Crucially, we extend these insights by demonstrating dose-specific effects: while high-dose methylprednisolone excels in symptom control, low-dose prednisolone provides superior vascular protection. This dichotomy may reflect differential impacts on endothelial repair pathways—a hypothesis warranting mechanistic investigation.

Wooditch et al. (Wooditch and Aronoff, 2005) reported that corticosteroids in initial KD treatment reduced coronary aneurysm incidence. Our findings align, showing that IVIG-plus-LDP therapy and IVIG-plus-HDMP therapies significantly outperformed IVIG-alone therapy in CAD prevention. Notably, IVIG-alone therapy ranked higher than MDMP-alone or HDMP-alone therapies for CAD reduction. Thus, children with CAD risk factors or established CAD at admission may benefit from IVIG-plus-LDP therapy, and corticosteroid monotherapy appears less effective than IVIG-based regimens. We should avoid corticosteroid monotherapy for CAD prevention. However, only one study evaluated MDMP-alone therapy for CAD incidence, limiting conclusions about this approach. Larger randomized controlled trials (RCTs) are needed to validate these findings.

After excluding the study by Ogata et al. (2012), the SUCRA value for IVIG-plus-LDP increased from 0.71 to 0.88, while that for IVIG-plus-HDMP decreased from 0.79 to 0.62. We interpret these shifts not as an invalidation of our primary analysis, but rather as evidence of effect modification by IVIG responsiveness status. The study by Ogata et al. (2012) enrolled a uniquely high-risk population with predicted IVIG resistance. Biologically, it is plausible that the optimal corticosteroid dosing strategy differs between patients who are predicted to be IVIG-responsive and those who are not. For example, while the overall analysis suggested superior performance of IVIG-plus-LDP, results after exclusion of the aforementioned study indicate that IVIG-plus-HDMP may be more effective in overcoming resistance in the high-risk, IVIG-resistant subgroup. This clinical heterogeneity is further supported by a reduction in between-study heterogeneity (τ) following the exclusion of this study. Notwithstanding this important nuance, the conclusions from our primary network meta-analysis remain robust and clinically relevant for the general population of Kawasaki disease patients, which was the primary focus of our review. The consistency of the findings across multiple prior distributions further strengthens the validity of our conclusions.

A seemingly paradoxical finding was that while IVIG-plus-HDMP was most effective in preventing initial treatment resistance, IVIG-plus-LDP ranked highest for preventing coronary artery abnormalities. This apparent discrepancy may be explained by effect modification. HDMP provides potent acute anti-inflammatory action, crucial for abating fever and preventing resistance in high-risk patients. In contrast, the anti-inflammatory effect of a LDP regimen may be more effective at suppressing the chronic vascular inflammation that leads to coronary complications in the broader population. This hypothesis is supported by sensitivity analysis, which showed that the superiority of HDMP for resistance was driven by studies enrolling high-risk, IVIG-resistant patients.

Corticosteroid-related side effects, including bradycardia and hypertension, were common in our analysis, consistent with prior studies. Goto et al. (2015) noted adrenal suppression with IVIG plus prednisolone, while Nagakura et al. (2017) reported bradycardia associated with IVIG responsiveness. Miura et al. (2005), observed increased sinus bradycardia, hypothermia, and hyperglycemia with high-dose corticosteroids, though hypertension rates were similar across groups. Importantly, these side effects were transient, mild, and reversible upon treatment cessation, supporting the safety of individualized corticosteroid regimens. Shulman and Rowley (2015) highlighted that corticosteroids as adjunctive therapy rapidly reduce fever and inflammatory markers, a finding corroborated by our study, where IVIG plus corticosteroids outperformed IVIG-alone therapy for antipyretic effects. The dose-dependent anti-inflammatory effect of corticosteroids suggests that higher doses achieve faster fever resolution.

## 5 Limitation

This meta-analysis has several limitations. First, the limited number of included RCTs restricts the robustness of our conclusions. Future well-designed RCTs with larger sample sizes are needed to confirm these findings. Second, only one study evaluated MDMP-alone therapy for CAD incidence, precluding definitive conclusions about this treatment. An updated meta-analysis incorporating additional studies on MDMP-alone therapy is warranted.

## 6 Conclusion

For the general KD population, IVIG-plus-LDP appears to be the most effective regimen for reducing resistance and CAD risk. However, the treatment hierarchy may be modified by IVIG responsiveness status, suggesting that high-risk patients may derive greater benefit from more intensive steroid dosing. This warrants verification in future stratified trials.

## Data Availability

The original contributions presented in the study are included in the article/Supplementary Material, further inquiries can be directed to the corresponding authors.
