# Supplementary material for: Comparison of multiple doses of corticosteroids in Kawasaki disease: a Bayesian network analysis
Source: Front Pharmacol. 2025 Oct 27;16:1661380. doi: 10.3389/fphar.2025.1661380 (PMC12598507; doi:10.3389/fphar.2025.1661380)
Supplement: Supplementary file 1 [file Table1.docx]

**1. PubMed (NCBI)**
**Search Strategy:**

1.1 "Mucocutaneous Lymph Node Syndrome"[Mesh] OR "Kawasaki Disease"[tiab] OR "Kawasaki Syndrome"[tiab] OR "Kawasaki's Disease"[tiab]

1.2 "Steroids"[Mesh] OR "Methylprednisolone"[Mesh] OR "Prednisolone"[Mesh] OR "Adrenal Cortex Hormones"[Mesh] OR "Glucocorticoids"[Mesh]

1.3 steroid*[tiab] OR corticosteroid*[tiab] OR glucocorticoid*[tiab] OR methylprednisolone[tiab] OR prednisolone[tiab] OR prednisone[tiab]

1.4 #2 OR #3

1.5 "Randomized Controlled Trial" [Publication Type] OR "Controlled Clinical Trial" [Publication Type] OR randomized[tiab] OR randomised[tiab] OR placebo[tiab] OR randomly[tiab] OR trial[tiab] OR groups[tiab]

1.6 #1 AND #4 AND #5

1.7 Filters: from inception - 2024/12/31

1. **EMBASE (Ovid)**
   **Search Strategy:**

**2.1 exp Kawasaki Disease/ OR ("Kawasaki Disease" OR "Kawasaki Syndrome" OR "Mucocutaneous Lymph Node Syndrome").ti,ab,kw.**

**2.2 exp steroid/ OR exp methylprednisolone/ OR exp prednisolone/ OR exp glucocorticoid/**

**2.3 (steroid* OR corticosteroid* OR glucocorticoid* OR methylprednisolone OR prednisolone OR prednisone).ti,ab,kw.**

**2.4 2 OR 3**

**2.5 exp randomized controlled trial/ OR exp controlled clinical trial/ OR random.ti,ab,kw. OR placebo.ti,ab,kw. OR trial.ti,ab,kw. OR groups.ti,ab,kw.**

**2.6 1 AND 4 AND 5**

**2.7 limit 6 to yr="1980 - 2024" [Adjust based on your database's actual date range filter]**

**3. Cochrane Central Register of Controlled Trials (CENTRAL, Wiley)**
**Search Strategy:**

**3.1 [mh "Mucocutaneous Lymph Node Syndrome"] OR ("Kawasaki Disease" OR "Kawasaki Syndrome"):ti,ab,kw**

**3.2 [mh Steroids] OR [mh Methylprednisolone] OR [mh Prednisolone] OR [mh Glucocorticoids]**

**3.3 (steroid* OR corticosteroid* OR glucocorticoid* OR methylprednisolone OR prednisolone OR prednisone):ti,ab,kw**

**3.4 2 OR 3**

**3.5 1 AND 4**

**3.6 Publication Date to 2024/12/31**
